# A method and app for measuring the heterogeneous costs and benefits of justice processes

**DOI:** 10.3389/fpsyg.2023.1094303

**Published:** 2023-05-16

**Authors:** Matthew Manning, Gabriel T. W. Wong, Christopher Mahony, Anushka Vidanage

**Affiliations:** ^1^City University of Hong Kong, Kowloon, Hong Kong SAR, China; ^2^Centre for Social Research and Methods, College of Arts and Social Sciences, Australian National University, Canberra, ACT, Australia; ^3^World Bank Group, Washington, DC, United States; ^4^Software Innovation Institute, College of Engineering and Computer Science, Australian National University, Acton, ACT, Australia

**Keywords:** justice reform, cost–benefit analysis, machine learning, data science, justice processes, heterogeneity

## Abstract

Can the impact of justice processes be enhanced with the inclusion of a heterogeneous component into an existing cost–benefit analysis (CBA) APP that demonstrates how benefactors and beneficiaries are affected? Such a component requires: (i) moving beyond the traditional cost benefit conceptual framework of utilising averages; (ii) identification of social group or population-specific variation; (iii) identification of how justice processes differ across groups/populations; (iv) distribution of costs and benefits according to the identified variations; and (v) utilisation of empirically informed statistical techniques to gain new insights from data and maximise impact to beneficiaries. In this paper, we outline a method for capturing heterogeneity. We test our method and the CBA online APP we developed using primary data collected from a developmental crime prevention intervention in Australia. We identify how subgroups in the intervention display different behavioural adjustments across the reference period revealing the heterogeneous distribution of costs and benefits. Finally, we discuss the next version of the CBA APP, which incorporates an AI-driven component that reintegrates individual CBA projects using machine learning and other modern data science techniques. We argue that the APP, enhances CBA, development outcomes, and policy making efficiency for optimal prioritization of criminal justice resources. Further, the APP advances policy accessibility of enhanced, social group-specific data illuminating policy orientation for more inclusive, just, and resilient societal outcomes.

## 1. Introduction

Intergovernmental organisations and leading international financial institutions cite grievances surrounding social group-specific exclusion from access to justice and security. For example, the joint United Nations-World Bank flagship report, “Pathways for Peace” (United Nations and World Bank, 2018) highlight the above as one of four arenas of social contest that inform the risk of violence.^1^ The World Bank has developed analytical tools for identifying the political economy and efficacy of governance and justice reform including the relationship between justice sector policy and programmatic approaches and the World Bank’s twin goals of reducing extreme poverty and driving shared prosperity (The World Bank, 2022).

To improve access to justice and the necessary evidence base to achieve this, a comprehensive economic framework for tracing how justice processes or the substantive consequences of justice processes vary across individuals and communities is crucial. Currently, however, measurements of benefits are not disaggregated, and cost–benefit analysis (CBA) typically relies on the average treatment effect (ATE), which does not adequately unpack the contextual variations that moderate outcomes across affected communities or sub-groups within communities.

This paper, therefore, considers the question: can the impact of justice processes be enhanced with the inclusion of a heterogeneous component into an existing CBA APP that demonstrates how benefactors and beneficiaries are affected? The development of such a framework requires: (i) moving beyond the traditional cost benefit conceptual framework of utilising averages (i.e., ATE); (ii) identification of variation or differences between groups or populations (e.g., individual actor attributes, political structures, institutions); (iii) identification of how justice processes differ across groups/populations; (iv) distribution of costs and benefits according to the identified variations (i.e., heterogeneous impact specific to the nature of justice policy/intervention or the context in which the policy/intervention occurs); and (v) utilisation of empirically informed statistical techniques to gain new insights from data and maximise impact to beneficiaries. Individually, these points are not novel, but it is their integration that provides new empirical opportunities.

The Pathways for Peace report takes important steps in identifying the delivery of education, health care, water, sanitation, justice and security as “the glue” binding state and society (Milliken and Krause, 2002) via the basic minimum citizens’ expect in order to accept state authority (Gilley, 2009). However, the report acknowledges the complexity of the relationship between service delivery and legitimacy (Brinkerhoff et al., 2012; Sacks and Larizza, 2012; Stel and Abate, 2014; Mcloughlin, 2015; Fisk and Cherney, 2017). State legitimacy depends on public expectations, which are informed by prior experiences (Nixon et al., 2017), geography, identity, and culture (Sturge et al., 2017). The report acknowledges the Sustainable Development Goals (SDG 16.3) to promote the rule of law and ensure equal access to justice for all (United Nations and World Bank, 2018). The report emphasizes that development approaches must identify *how*, not only *why*, justice processes and outcomes discriminate, or are perceived to discriminate, against certain groups (United Nations and World Bank, 2018). Beyond establishment of frameworks identifying how processes treat groups differently, development actors must grapple with how to most equitably deploy finite resources to change that unequal treatment. Decisions regarding the equitable deployment of finite resources may be influenced by static (e.g., ethnicity) and dynamic (e.g., socio-economic status) indicators that recognise group differences within a community. Incorporating these data into CBA enables identification of how justice processes treat different groups and the consequences (economic and social) for those groups.

To take forward a framework that also appropriately prioritizes the use of finite resources, the costs and benefits of policy options must be properly weighed within the context of the “…dynamic interaction of actors, institutions, and structural factors over time” (United Nations and World Bank, 2018). CBA tools necessary to achieve this outcome must drive optimal justice sector decisions in a systematic, data-informed, and resource-efficient way, including drawing on a range of analytical products. These products include, for example, indexes that incorporate indicators of group differences, legal needs surveys, and justice for the poor dispute resolution analytics. By drawing together data from analytical processes, scope for comparison of various justice-related datasets is enabled.^2^ Most importantly, the analytical approaches assist in identifying the various elements of justice processes whose costs and benefits may then be traced. Such empirical approaches that trace the relevant costs and benefits that shape justice processes require economic analysis (EA), which is often undertaken using CBA tools. Early CBA tools such as the Washington State Institute of Public Policy’s (WSIPP) Benefit-Cost Tool (Aos and Drake, 2010) and the Manning Cost Benefit Tool (MCBT) [Version 1 published by College of Policing UK (2016)], were developed to support an evidence-based approach.

More recent developments have been undertaken by the authors of this paper (Manning et al., 2022), representing an extension of the above-mentioned MCBT, which begin to incorporate machine learning and artificial intelligence, including the development of an online CBA APP (Manning et al., 2022) as showcased in Manning et al. (2018). This APP takes important steps towards robust and time-sensitive analytical methods. The online CBA APP (currently in various stages of development), has been validated using a range of crime data,^3^ providing a framework with systematic data management capacity that enables user input support and EA. The online APP also includes a new heterogeneous component (which we describe and test here), that reveals and measures variations across social groups informing justice reform investment decisions that best manage and mitigate social group specific grievances while maximising economic consequence to society. We refer to this APP hereon as the “enhanced CBA APP”.

The first question is to what extent the enhanced CBA APP selects the most efficient option/s available while accounting for heterogeneous treatment effects? Traditionally, CBA focuses on average benefits (e.g., ATE) and average costs. These metrics are not always adequately disaggregated among benefactors and beneficiaries. Heterogeneous treatment effects and/or costs are most often present but unaccounted for. The retention of big data enables greater capacity to undertake more “comprehensive”, rather than “narrow” CBA. This means that efficiency potential is hidden in both the efficacy and implementation of public and private programs, if policies can be targeted at those who, net of costs, benefit the most and/or are most vulnerable. In this paper, we speak in terms of disproportionate social group access to public goods including access to justice (United Nations and World Bank, 2018).

We recognise that the literature on justice sector-specific CBA is developing, with much scope for advance as the data illuminating human behaviour becomes more representative, reliable, and accessible. Sen (2000) notes the historically narrow nature of analysis solely through market values, which omit social choices that enable freedom of valuation and increased informational inputs. Harley et al. (2019) define narrow CBAs, which they observe as commonly employed, as comprising “direct tangible benefits and costs” (p. 16) resulting from the programmatic or policy change.^4^ Harley et al. define “comprehensive” as direct tangible benefits and costs plus “a more extensive accounting of the indirect economic benefits to all those affected,” including “benefits to individuals, the justice system, the economy and society” (p. 16).

Diverse datasets that capture heterogeneity, when drawn upon via established statistical techniques, empower data management systems and development practitioners to deliver more comprehensive CBAs. In this context, a CBA needs to recognise and respond to the logic of how social groups cope individually and collectively in specific settings. Existing measures of access to justice (e.g., the World Justice Project Rule of Law Index) (The World Justice Project, 2019) alongside data collected by governments and development actors, may be incorporated into a CBT such as the enhanced CBA APP. Using CBTs that incorporate heterogeneous components is important to determine how resources directed to eliminate barriers to justice can be efficiently and effectively allocated.

Considering the knowledge and gaps outlined above, here we: (i) outline a method for capturing heterogeneity based on earlier empirical studies undertaken on this issue; and (ii) demonstrate the method and enhanced APP using primary data from a study conducted in 2006 on the costs and outcomes associated with a developmental crime prevention intervention undertaken in Australia; (iii) apply our method to develop CBA outcomes that incorporate the heterogeneous distribution across intervention subgroups; (iv) discuss how the method we developed in this paper is incorporated into the enhanced CBA APP; (v) discuss future developments of the enhanced CBA APP that will incorporate modern data science techniques. Overall, the method we describe, test, and apply to the enhanced CBA APP can capture the complexity of prevention and will assist policy makers in producing evidence sensitive to contextual social group-specific variation.

## 2. A method for capturing heterogeneous costs and benefits across groups

The typical purpose of CBA in the context of justice is to developed evidence on the economic viability of publicly or privately funded policies/programs/interventions. As briefly highlighted above, the benefits identified and measured are based on parameter estimates of the average effect of a given access to justice program or intervention, which are then compared to the associated average costs (Boardman et al., 2017). However, as revealed above, substantial heterogeneity (including levels of access) often exists in the benefits and/or costs across individual and societal levels. As such, the traditional CBA approach may hide valuable information about which social groups benefit the most net of costs.

The Kaldor–Hicks efficiency criterion is commonly applied in CBA and states that a publicly funded program can be justified when the overall benefits outweigh the costs. This criterion is in contrary to the earlier Pareto criterion. It requires that no one will suffer from any change under the proposed intervention conditions whereby the Kaldor–Hicks criterion identifies the possibility for compensation. Theoretically, a program under the Kaldor–Hicks efficiency criterion can be defended even if it produces undesirable or negative outcomes for some individuals or groups, so long as the overall benefits are greater than the overall costs to society (Manning, 2008; Boardman et al., 2017). However, we propose that the conceptual foundation of CBA (Kaldor–Hicks Criterion) does not adequately address the issue of heterogeneity in impact such as access to justice. We argued that an important consequence of accounting for heterogeneity is that international development assistance-driven justice sector gains, net of costs, may disproportionately target certain population subgroups. This argument rationalizes the method we discuss below.

### 2.1. Average and quartile treatment effects

The availability of data now makes obtaining average treatment effect (ATE) easier, particularly when random control trials (RCT) are employed. In short, the average treatment of the treated group (ATT) equals the ATE (for the population). The RCT lends itself to a simple nonparametric analysis of the treatment effect, given by

$$ATT=\mathrm{ATE}={\bar{Y}}_{1}-{\bar{Y}}_{0}$$

where, ${\bar{Y}}_{1}$ is, for example, the average number of episodes of political violence [for example, using the Armed Conflict Location and Event Database (Raleigh et al., 2022)] for the experimental group, while ${\bar{Y}}_{0}$ is the average number of episodes of political violence for the control group. The average effect of the intervention of interest is simply the difference between the two averages. As stated above, however, the shortcoming of ATE is our lack of understanding of the distribution of effects. In addition, the mechanisms that make the prevention of conflict (economically) successful.

It is inherently difficult to measure the policy impact for each individual, since, by definition, the counterfactual for each individual under the policy may be unobserved depending on the possibility for appropriate matching of experimental and control groups. Often, even when these groups are matched, the heterogeneity of policy impact is buried in the average effect. We can, however, disaggregate the population based on what may be described as moderators or diversity variables (e.g., age, gender) and compute the distribution of outcomes within the population (Heckman et al., 1997). A sign of heterogeneity is revealed by an uneven distance across individuals. Although we cannot fully appreciate the distribution of effects, we can nevertheless gain useful knowledge about whether there exist heterogeneous effects across groups in the population.

For example, a defensive policy aimed at reducing domestic terrorism and violence may have significant distributional costs and benefits across the population and, therefore, relying on the ATE could overestimate the effects on some in the population but underestimate the effects on others. So, if one of our variables of interest was social dominance orientation (SDO) (intellectualised as an individual’s preference for inter-group hierarchies within a social system or group-based discrimination), we must capture the levels of SDO that exist across our population. We note here that individuals with high SDO are more likely to support public policies that promote or recreate social hierarchies, while people with low SDO favour more equality-based policies (Pratto et al., 1994; Vorsina et al., 2019). Other variables may exist, other than SDO, which moderate the policy impact on different population subgroups. These also serve as potential moderators that require examination. Exploring the heterogeneous distribution based on these moderators will allow for a comprehensive understanding of the economic implications of policy decisions. The question now is, how can we disaggregate the population according to the observed and sometimes unobserved differences?

### 2.2. Quantile treatment effects

There exist several methods to capture heterogeneous effects. To capture such variation we outline, in what is a standard approach, the method used by Kristensen et al. (2017). The authors apply a method that, like us, attempts to monetize the economic benefits associated with an intervention. In addition, the authors clearly outline how they overcome the shortfalls of traditional CBAs, which focus exclusively on ATEs. In this study the authors employ a randomized control-trial experiment to examine the heterogeneous impact of the Danish return-to-work program. The authors successfully demonstrate: (i) the benefits of estimating quantile treatment effects (QTE) (a well-established technique used widely in the social sciences and included in a range of statistical programs^5^); and (ii) the use of the efficiency potential (what we call potential efficiency gain) to assess the net benefits resulting from the reallocation of resources sensitive to the QTE. Kristensen and colleagues, based on their results, propose a screening system to triage participants to create efficiencies and ultimately maximise overall program effects by considering subgroup differences.

Moving beyond Kristensen and colleagues, we introduce a method for classifying and estimating variability in effect among groups (either categorically or continuously) and the inclusion of this method (outlined below) into the enhanced CBA APP. Categorical divisions of the population could be based on some naturally formed social groups (e.g., ethnicity and gender). The impact of a policy as outlined in our literature review may be moderated by these factors where the policy may unevenly affect different groups within the population. For continuous latent variables (e.g., level of SDO), which are measured along a continuum, we could use confirmatory factor analysis (CFA) to study and describe the relationships between a set of observed variables (e.g., based on Likert scale items) and the specific set of continuous latent variables of concern (Fox, 2010; van der Linden, 2016). This is especially helpful when no pre-established indexes are available. This allows us to create a combined measure capturing the level or degree of a previously unobserved latent characteristic or factor. Back to our SDO example, when there are no reliable indicators that accurately classify individuals in the community with respect to level of SDO, an evaluation team may decide to incorporate certain assessment criteria (measured continuously) and assess the uniformity of these criteria in measuring SDO levels (the previously unobserved latent variable). Following Bitler et al. (2006), consider two distributions where we have our experimental and control groups, respectively, *F*_1_ and *F*_0_, and define quantile treatment effects (QTE) as $\Delta_{q}=y_{q}\left(1\right)-y_{q}\left(0\right)$ , where $y_{q}\left(t\right)$ is the *q*-th quantile of distribution *F_t_*.

### 2.3. Accounting for heterogeneity

**Figure 1:**
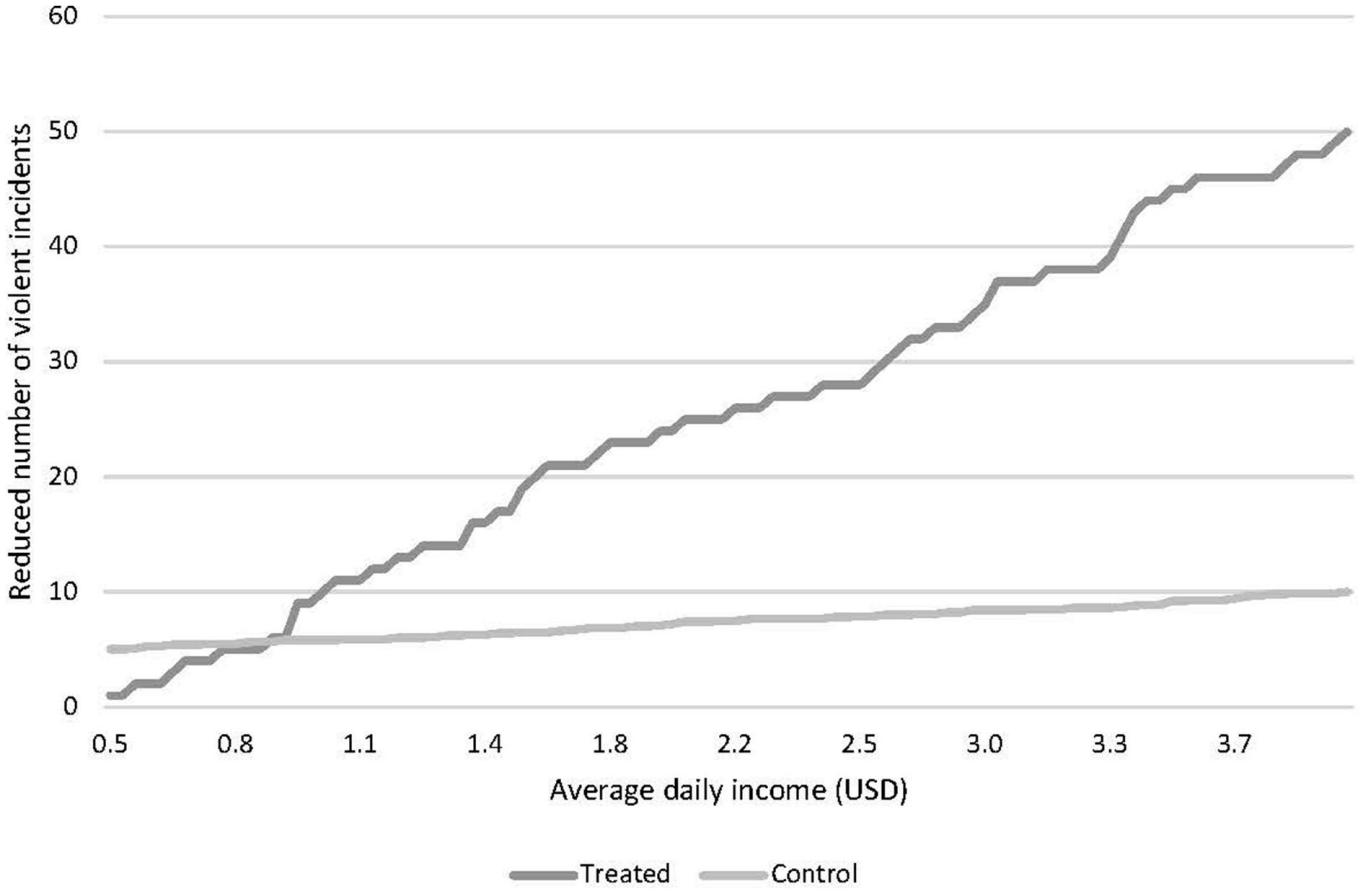
Distribution of number of violent incidents, treatment and control groups.

Policy outcome differences within the distribution of the continuous latent variable will indicate whether the outcome effects are stronger for some individuals than for others. Figure 1 shows the cumulative distribution for hypothetical treatment and control groups in an RCT of a given policy.

**Figure 2:**
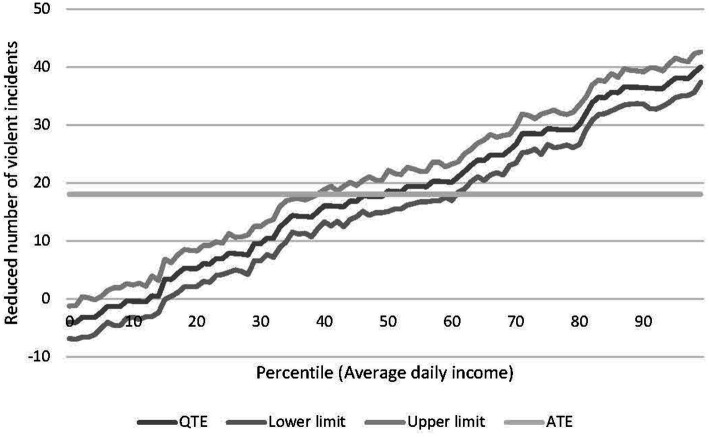
Average quantile treatment effects.

The horizontal difference in the distributions indicates the impact of the policy, and this difference corresponds to the QTE shown in Figure 2. A horizontal line for the ATE is presented in Figure 2, which shows no difference across the continuum. Figure 2 clearly indicates that the hypothetical policy has varying impacts across the distribution. Given the positive relationship between the policy impact and the latent continuous variable, positive effects are found from about the 10th percentile onward. The comparison between ATE and QTE, shown in Figure 2, reveals substantial heterogeneity in the treatment effect.

**Figure 3:**
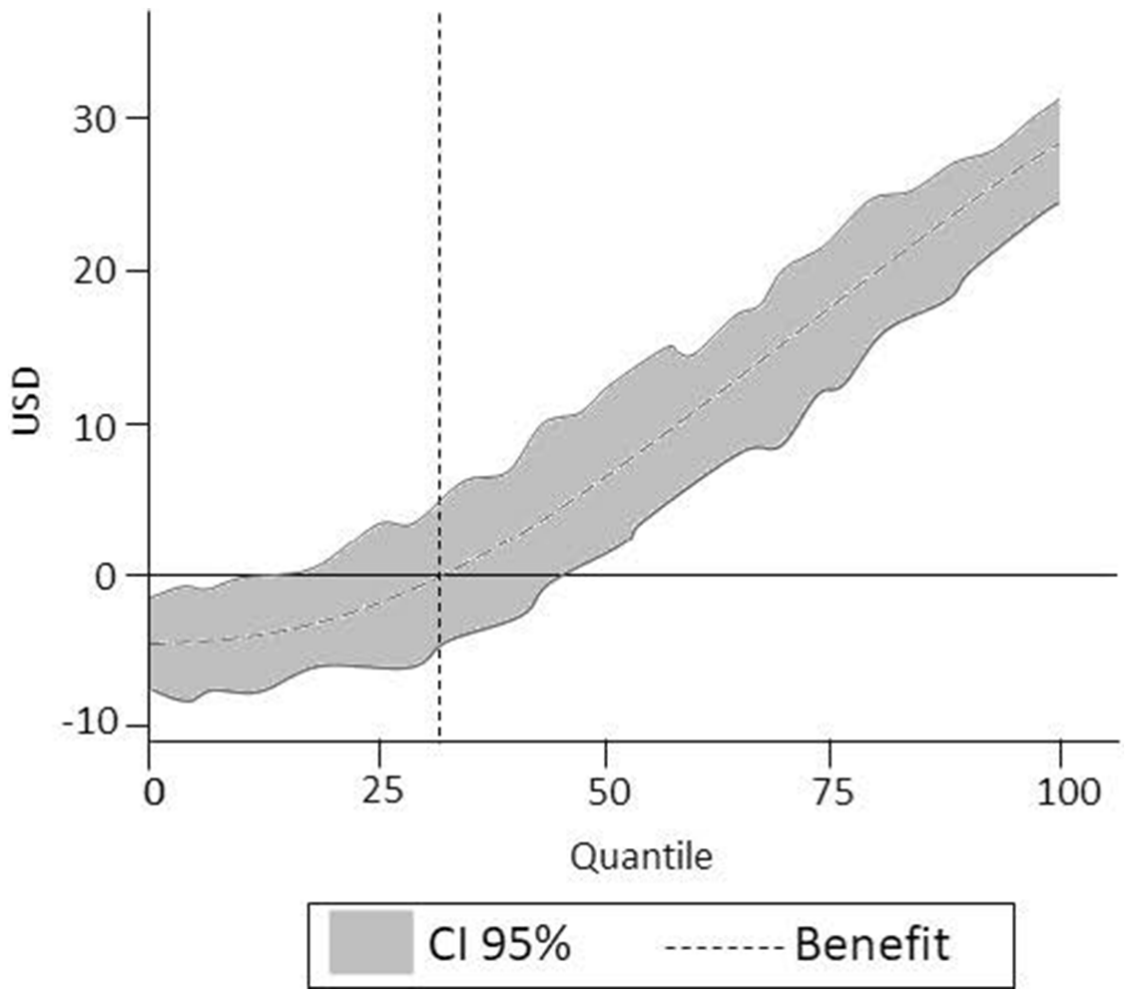
Quantile treatment effects and break-even analysis.

If we were able to perfectly screen individuals and only allocate treatment to individuals with a treatment effect above the average cost level, (in the absence of individualized cost data), that is individuals above the break-even line shown in Figure 3, then, we would be able to improve the overall net benefit of the policy. In this example, there is room to improve the net benefit of the policy for the population concerned. We define this as the potential efficiency gain (PEG). That is, the increased benefits net of costs that would accrue to society, if it is possible to perfectly screen individuals to those who benefit at least as much as the costs involved.

PEG is given by:

$$\mathrm{PEG}=\frac{\left(BC\;\mathrm{maximised}-BC\;\mathrm{baseline}\right)}{BC\;\mathrm{baseline}}$$

where “BC maximized” refers to the scenario where the benefit–cost ratio is maximized due to a selective application of treatment/policy (i.e., treatment only applied to those individuals with a positive net benefit), and “BC baseline” refers to the baseline where the treatment/policy has been adopted to everyone as a universal treatment option. The reader should note that there may be heterogeneity in benefits across individuals not captured by the quantile methods employed—this may be a result of variation of policy impact based on other unobserved moderators in the population. Hence, the PEG is not a maximum benefit that might be obtained via controlling application of treatment/policy to certain groups based on a limited number of observed moderators.

### 2.4. Examples of moderators that expose heterogeneity in access to justice

The PEG can only be calculated if evaluators can detect and identify the key factors, whether they are static or dynamic, categorical, or continuous, that moderate the policy impact. As such, one needs to identify which covariates drive the heterogeneous effect. One could, for example, interact the intervention with a selection of theoretically relevant covariates (e.g., SES, level of education). If we find that the interaction terms are not statistically significant, then the heterogeneity observed earlier might not be due to these factors/potential moderators. Of course, the method employed will be dependent on the quality of these data and the hypotheses being tested, but here we only provide one of many methods to capture heterogeneity and estimation of PEG using an observed moderator.

Given that perceptions of exclusion from access to justice are associated with increased risk of violence (as discussed above), “exclusion” may constitute a covariate that moderates policy impact. For example, the World Bank recently concluded the establishment of the World Wide Exclusion Indicators (WEI) (The World Bank, 2021a), which may be employed as data indicating selectivity of the provisional benefits (and costs). The WEI represent, across 181 countries from 1900–2018, denial of individuals’ access to services or participation in governed spaces based on their identity or belonging to a particular group. The indicators measure exclusion across four dimensions: exclusion from civil liberties; exclusion from access to public services; exclusion from access to state jobs; and exclusion from access to state business opportunities. Exclusion in these dimensions is grouped by five indices: socio-economic group; by gender; by rural/urban location; by political group; and by social group (including caste, ethnicity, language, race, region, religion, migration status, or some combination thereof). The WEI, therefore allow for exclusion, for example by gender, within a country or across a region, for example the Middle East across the four dimensions (Pillai, 2019). Of relevance to justice processes are the dimensions of exclusion of social groups in their access to civil liberties and the state service of justice. However, the efficacy of equal access to other state services, to state jobs, and state business opportunities may inform, in particular, identification of social group-specific exclusion in CBA modelling of justice processes to resolve equal access in those dimensions. It is these dimensions, for example, that provide the data necessary to examine the distribution of effect and expose heterogeneity.

A further covariate example that may moderate policy impact may be “governance”. The World Bank’s Worldwide Governance Indicators (WGI) (The World Bank, 2021b) reports aggregate and individual governance indicators for over 200 countries and territories from 1996, for six governance dimensions: Voice and Accountability; Political Stability and Absence of Violence; Government Effectiveness; Regulatory Quality; Rule of Law; and Control of Corruption. These aggregate indicators, based on over 30 individual data sources, combine the views of many enterprise, citizen, and expert survey respondents.

As data emerges specific to *how* different justice processes exclude social groups, frameworks can be developed and vetted to inform what constitutes optimal social (such as inclusion) and economic net benefits. The United Nations and World Bank, on this issue specific to processes of accountability for atrocity crimes, note:

“Weighing the equality of accountability processes against the imperative to bring perpetrators to book is critical to the challenge of advancing stabilization and justice in conflict-affected environments under SDG 16. Accountability processes may exacerbate grievances related to specific social groups if they are perceived to discriminate between groups. How and why the real or perceived unequal treatment of social groups actually occurs varies from one process to another. Frameworks to identify how accountability processes treat groups differently can help to identify ways in which to pre-empt spoilers and mitigate risks of conflict.” (United Nations and World Bank, 2018, pp. 167–168)^6^

## 3. Method

In this paper we use the enhanced CBA APP to process the cost and benefit data. We adopt the QTE method as described above. The enhanced APP proposes to overcome the shortcomings of existing cost–benefit tools [e.g., the Manning Cost–Benefit tool versions 1 and 2 (Manning et al., 2016; Manning and Wong, 2016a,b)], with the inclusion of the heterogeneity component which allows us to: (i) identify variation or differences between groups or populations (e.g., individual actor attributes, political structures, institutions); (ii) identify how justice processes differ across groups/populations; and (iii) distribute costs and benefits according to heterogeneous impact.

**Figure 4:**
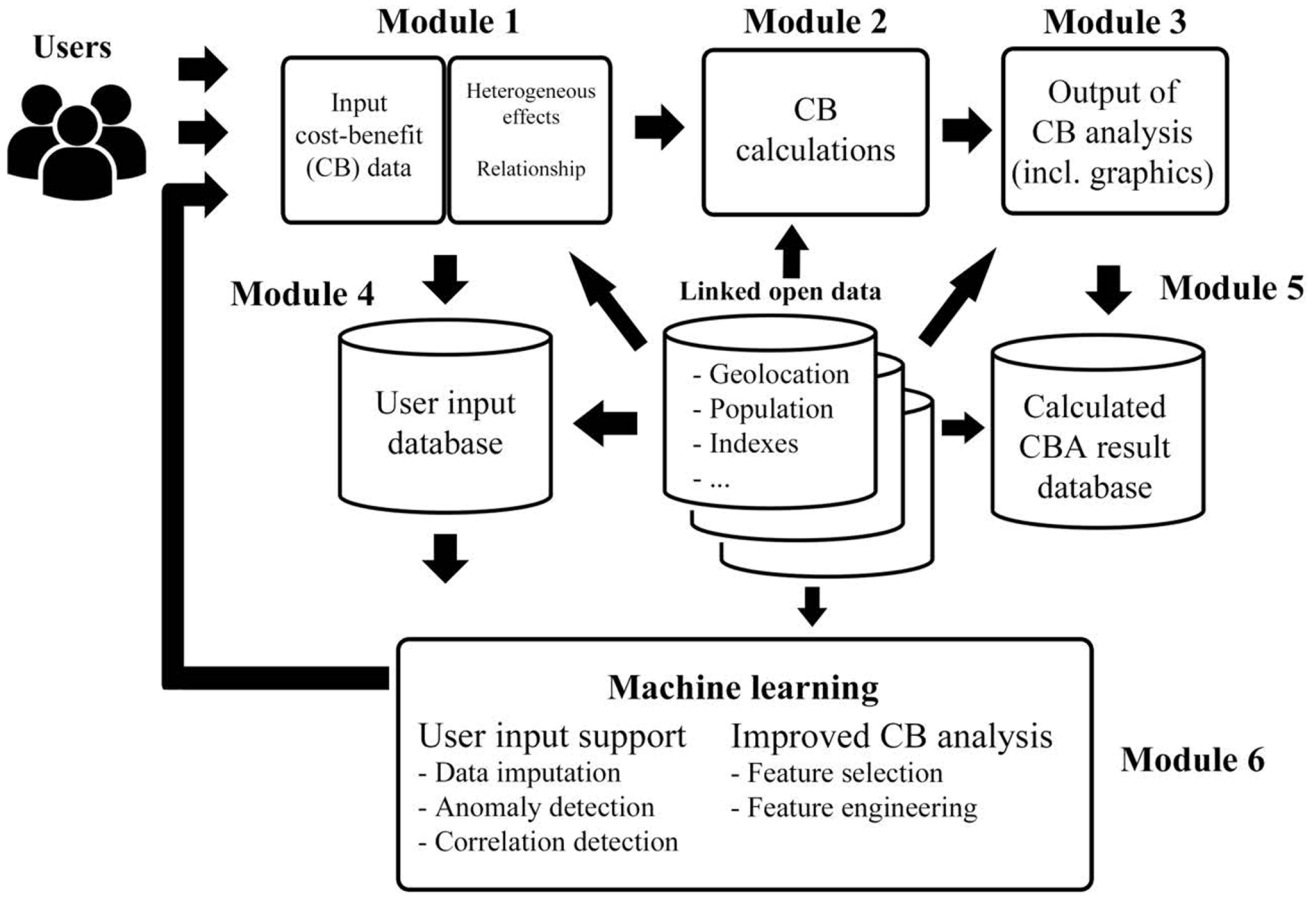
An enhanced CBA APP.

Figure 4 illustrates the enhanced CBA APP. In this study, we demonstrate three of the six interacting modules (Modules 1, 2, and 3). A full discussion of the six modules included in the enhanced CBA APP is provided by Manning et al. (2018).

In terms of *Module 1*, costs can be disaggregated by bearer (e.g., criminal justice system, local government, business, and society) and the outcomes (either positive or negative) can be, as described above, affected by identified moderators. Here, the user of the enhanced CBA APP specifies the identified and observed moderators (i.e., continuous or categorical). If the variable is continuous, the evaluator can conduct correlational analyses to justify the use of specific characteristics or factors of the population as a moderator. If the variable is categorical, then the evaluator can conduct ANOVA to identify the association between the moderator and the outcome of interest. Once the relationship between the moderator/s and the outcome is established, the benefits side of the CBA can now account for the heterogeneous effect.

This takes us to *Module 2* where CBA calculations are performed. Here we include steps such as accounting for: (i) economic assumptions (i.e., inflation and discount rates); (ii) confidence intervals (i.e., worst and best-case scenario); (iii) optimism/confirmation bias correction; (iv) percentage of total cost borne and spent each year; (v) attributable fraction (i.e., percentage or proportion of costs attributed to the intervention of concern); and (vi) heterogeneous effects on outcome. *Module 2* allows the evaluator to separate outcomes across affected social groups (e.g., beneficiaries) if the moderator is categorical in nature (e.g., SES). If the moderator is continuous in nature, then the evaluator can employ a range of methods, for example QTE as described above, to generate patterns of benefits across the distribution of individuals or groups along the continuum.

In *Module 3*, the enhanced CBA APP provides the outputs of the economic analysis. In summary, the APP allows for cost-feasibility analysis (i.e., comparing the overall costs of the project against the budget), cost-savings analysis (i.e., comparing the costs of the project against the savings generated from avoided crimes), cost-effectiveness analysis (i.e., comparing the costs of the project against the number of units of output such as the number of crimes prevented) and CBA (i.e., comparing the costs of the project against the overall benefits—avoided crimes and other benefits such as enhanced safety). Tables and plots are also included to display, for example, net costs, net benefits, cost–benefit ratio, net benefits by bearers and potential efficiency gain (PEG—as described above).

### 3.1. Data employed to test modules 1 to 3 using the enhanced CBA APP

We employ data from the Pathways to Prevention Project (Homel et al., 2006) to test *Modules* 1 to 3 of the enhanced CBA APP. The Pathways to Prevention project was established over twenty years ago as an early intervention, developmental crime prevention initiative focused on the transition to school in one of the most disadvantaged urban areas in Queensland, Australia (Homel et al., 2001). Here, the goal was to provide positive pathways to individuals at-risk of later learning and behavioural problems that may eventually lead to crime and deviance. The project comprised two components: (i) a preschool intervention program (PIP), which was a child-focused and school-based set of activities; and (ii) a family independence program (FIP), which was a family-focused and community based set of activities run by Mission Australia (Manning et al., 2006).

In this paper, we focus exclusively on the PIP component. PIP aimed to enhance the participant’s readiness to succeed at school. Through a sequence of structured small-group interactions with either specialist teachers or program staff, PIP aimed to develop a participants: (i) communication skills by introducing more abstract language, complex vocabulary and appropriate grammar formats as part of the preschool experience, and (ii) social skills to improve a participants ability to better interpret social interactions, overcome emotions that are unproductive (e.g., anger and anxiety) and develop strategies for dealing with problems that often occur during exchanges with peers (Manning et al., 2006).

To undertake our CBA, we use cost data derived from Manning et al. (2006) and outcome data collected from the chief investigator of the project, Professor Ross Homel. Project costs were estimated separately for three distinct stages: development, implementation, and evaluation. The cost analysis method employed and cost estimates are described in the study undertaken by Manning et al. (2006). Part of our CBA also requires us to estimate potential costs of a negative outcome—in this case the cost of dealing with behavioural problems—or the avoided costs if the intervention produces outcomes that reduce the probability of a child needing future behavioural management intervention.

Manning et al. (2006) estimated the costs of four behavioural management alternatives (grouped into two categories) that may be required if a child presents with behavioural problems in the early years of education. Category 1 consists of programs developed to help improve the behaviour of children with borderline or less challenging behavioural problems, while category 2 consists of programs aimed at helping those children with more severe or extreme behavioural problems (see Table 1). Homel et al. (2015) measured difficult and challenging behaviour using the Rowe Behaviour Rating Inventory (RBRI). The RBRI is a validated teacher checklist used to assess the level of children’s difficult behaviour (Rowe and Rowe, 1995). In this paper we classify children who received a score of 20 to 29 as displaying a low level of poor behaviour and thus only requiring little assistance (i.e., Alternative 1). We coded children receiving a score greater than 30 but less than 40 as requiring more assistance (i.e., Alternative 2). Children who received a score greater than 40 were identified as having more severe or extreme behavioural problems and fell into Category 2 type programs (RBRI 40–49—Alternative 3; RBRI ≥ 50—Alternative 4).

**Table 1:** Behavioural management programs.

Category 1	
Alternative 1	Pathways Communication Program (RBRI score 20–29)
Alternative 2	School district behavioural management teams (Inala Cluster)—known as Behaviour Support Team, Corinda District (RBRI 30–39)
**Category 2**	
Alternative 3	Pathways Social Skills Program (RBRI 40–49)
Alternative 4	Behavioural School (Tennyson Special School) (RBRI 50+)

The costs of the alternative programs estimated by Manning et al. (2006) are presented in Table 2.

**Table 2:** Cost of alternative behavioural management alternatives.

Intervention	Budget Cost	No. of participants	Per participant cost
Alternative 1	$47861.41	125	$382.89
Alternative 2	$236312.93	145	$1,629.74
Alternative 3	$13999.93	100	$139.99
Alternative 4	$417460.32	21	$19,879.06

### 3.2. Measuring heterogeneous impact of PIP

In terms of our analysis of potential heterogeneous outcomes, we use scores derived from the same data used by Homel et al. (2015). As described in brief above, RBRI forms a total measure of behavioural adjustment ranging from 12 (positive adjustment) to 60 (poor adjustment). In the longitudinal study undertaken by Homel et al. (2015) there were five measures of behavioural adjustment across the reference period, one for each academic year. Our model specifies behavioural adjustment for the fifth assessment period as the dependent variable, behavioural adjustment for the first baseline period as a control, and the exposure to PIP as an independent variable. The model also analyses the heterogeneous impact of PIP on behavioural adjustment through a series of regression analyses according to the number of siblings of the program participants (i.e., our diversity variable). We chose number of siblings as a diversity variable as it has been shown that children that have many siblings tend to display an increased odds of adverse developmental outcomes (de la Rochebrochard and Joshi, 2013).

In practice, then, compliance at Time 1 has been partialled out, leaving only the difference between Time 1 and Time 5 to be explained by the independent variable in the model (Cohen and Cohen, 1983). Using real data derived from Homel et al. (2015), we applied the *rnorm*() function of the R Studio (R. Studio Team, 2020) to simulate nine datasets, each with 1,000 samples, with a child having no siblings to 8 siblings. Our method ensures that the real data informs the simulation, ensuring parameters used for each simulated variable, including the RBRI scores and the PIP intervention, were as close to the real world as possible. For more details of the method employed please refer to Peng (2022).

## 4. Results

Our regression results show that PIP led to statistically significant poor behavioural adjusted outcomes for participants with no siblings (*β* = 10.45, *p* < 0.001), 5 siblings (*β* = 9.10, *p* < 0.001), 7 siblings (*β* = 12.27, *p* < 0.001) and 8 siblings (*β* = 10.83, *p* < 0.001) as revealed by the change in RBRI score post intervention. The PIP, however, was estimated to be beneficial to children with 1 (*β* = −7.00, *p* < 0.001) or 2 siblings (*β* = −2.09, *p* < 0.001), but had no statistically significant effect on children with 3 (*β* = 0.57, *p* > 0.1), 4 (*β* = 2.01, *p* > 0.1) or 6 siblings (*β* = −0.15, *p* > 0.1).

Based on these results, we further estimate the number of individual children who potentially may be triaged into the aforementioned alternative treatment and behavioural management programs (as described in Table 1). The difference in proportion of children with an RBRI score of 20–29 (category 1), 30–39 (category 2), 40–49 (category 3) and 50–60 (category 4) were observed between the intervention group (with PIP) and control group (without PIP). Such a difference in proportion was applied to the actual data collected by Homel et al. (2015), suggesting either a potential reduction or increase in the number of children who may require future behavioural management interventions. The estimated number of children who require additional behavioural management interventions is based on the percentage of children sorted into one of the four RBRI score categories before and after the intervention. This is calculated by applying the percentages derived from the simulated dataset to the actual number of participants in the Homel dataset. We present these results in Table 3 where we show, for example, that the estimated number of children requiring Alternative 1 (i.e., additional behavioural management intervention) with one sibling is 1.92, Alternative 2 [−2.44 (negative sign signifying a reduction in children requiring intervention)], Alternative 3 (−1.44), and Alternative 4 (−1.44).

**Table 3:** Estimated CBA results according to the number of siblings.

No. of siblings	Costs (AUD)	Benefits (AUD)	Net benefit (AUD)	Estimated no. of children requiring alternative 1	Estimated no. of children requiring alternative 2	Estimated no. of children requiring alternative 3	Estimated no. of children requiring alternative 4
0	$4611.92	−$20928.98	−$25540.90	−2.00	1.20	−1.00	1.00
1	$13835.76	$32070.15	$18234.39	1.92	−2.44	−1.44	−1.44
2	$28824.5	$102873.79	$74049.29	−7.42	−9.42	0.74	−4.27
3	$15565.23	$−	−$15565.23	—	—	—	—
4	$10376.82	$−	−$10376.82	—	—	—	—
5	$6917.88	−$27133.43	−$34051.31	−1.80	5.00	−1.40	1.00
6	$6341.39	$−	−$6341.39	—	—	—	—
7	$1729.47	−$2876.68	−$4606.15	−1.00	2.00	0.00	0.00
8	$4611.92	−$4646.32	−$9258.24	−1.00	3.00	1.00	0.00

We then applied the outcome data with the cost data described above into the enhanced CBA APP. Since the provision of PIP did not affect the RBRI score of some of the subgroups (non-statistically significant effect), no benefits could be attributed to those groups. As such, the economic benefits for these groups were not estimated (see Table 3 column 3, row 5). For other groups, however, we found positive and significant benefits (resulting in a positive avoided cost), and also negative and significant benefits (resulting in additional future treatment cost). The results presented in Table 3 show the number of children under each category (i.e., by number of siblings) multiplied by the corresponding per participant costs of the management programs to generate an estimate of the costs, benefits, and net benefits of PIP (i.e., the avoided costs of behavioural management). As discussed above, PIP was most beneficial for those participants that had 1 or 2 siblings. Table 3 reveals the net benefits to PIP participants with 1 or 2 siblings of $ $18,234.39 and $74,049.29, respectively.

**Figure 5:**
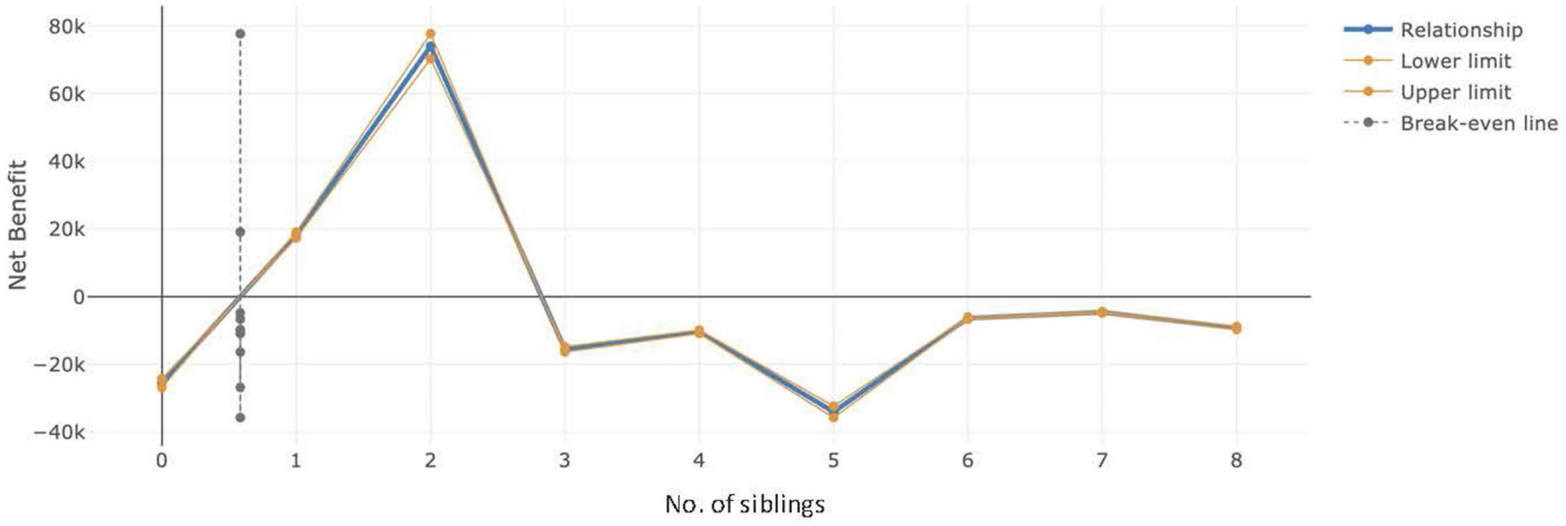
Comparison of net benefit according to the number of siblings.

The above economic estimates (derived from the enhanced CBA APP) are consistent with the findings of our regression analyses, both in strength and direction, where there were positive estimated benefits of the PIP intervention for children with 1 or 2 siblings and negative estimated benefits for children with no siblings or 5 or 7 or 8 siblings. Figure 5 below, which is a figure derived from the enhanced CBA APP, illustrates this relationship. The figure suggests that the PIP should only be strategically provided to children with 1 or 2 siblings to maximise its benefits and avoid any unintended adverse effect of the PIP towards children with different numbers of siblings.

We note that the example we use in this paper to illustrate to method and APP uses real data but simulates to adjust for small sample size and the potential outcomes of children requiring some form of future behavioural management intervention. In the absence of the above-described method that estimates the heterogeneous distribution of outcomes across the PIP intervention group and enhanced CBA APP (modules 1 to 3 of the APP), we would have found it difficult to fully appreciate: (i) how the benefits of the intervention are distributed across the intervention group participants; (ii) the avoided costs of PIP in terms of future behavioural management interventions; and (iii) the best targeting of resources among the intervention group (in this case children with 1 or 2 siblings) to enhance the economic efficiency of the intervention, which arguably may lead to potential improved economic returns on investment.

## 5. The benefits of the described CBA APP modules and next steps

Presented above was a clear outline and test of *Modules* 1 to 3 of the enhanced CBA APP. The data driven capacity within the current version of the enhanced CBA APP can identify which justice processes and societal factors are most significant for the costs and benefits of processes specific to context. The current APP, therefore, is capable of accounting for macro variables like inflation, provision of best and worst-case scenarios, identification and accounting of data bias, proportion of costs borne per year, and effects on outcome, including outcomes specific to social groups, to context and to specific intervention elements. Manning et al. (2018) provide a detailed discussion on these elements. However, the current version of the enhanced CBA APP is capable of more than what we have presented here. Below we describe three additional modules that are currently in various stages of development, testing and implementation.

*Module 4* (as shown in Figure 4) improves upon earlier economic tools, such as the MCBT, by drawing upon and exploiting machine learning (ML) techniques. Here, we incorporate a “User Input Database” module, where all data entered by users into the APP is stored as one set of records per project. The enhanced APP will allow the user to store their input data on their server, providing a single data resource for the ML module (i.e., *Module 6*). Here, we intend to provide source code to allow users to manage their data on their own server, utilising their own security protocols. Specifically, since *Module 1* identified and established a relationship between moderators and outcome, *module 4* then captures those relationships allowing the system to learn from these relationships and identify moderators that are relevant to outcomes concerned. This allows for the creation of a more comprehensive database and the possibility of subsequent ML, as described below in *Module 6* (e.g., imputation of missing data).

*Linked to Module 4 and Module 5* will provide code for users to build a database to store the calculated results *after* analysis. Storing the calculated results is crucial because it provides the ability to model the relationships between all input-relevant cost and benefit data (*Module 1*) and the output of CBA (*Module 3*). Therefore, the database in this module of the workflow diagram stores the benefits (that could also be weighted using a harm index^7^) and analysis data, enabling the system to map the relations between input and output, and exploit this to learn and improve CBA over time.

Finally, *Module 6* integrates ML techniques to achieve two main goals, namely: (i) to provide input support to the user by imputing missing values (currently in testing stage), identifying potentially erroneous values, and make suggestions about relevant contextual factors; and (ii) to improve the analytical capabilities of CBA by usefully reducing the number and types of variables to minimize user effort (e.g., time-consuming data entry) and develop better estimates (e.g., cost savings; crimes avoided), based on what the system learns from earlier projects.

## 6. Discussion and conclusion

The moral and commonly aspirational arguments for policy and programmatic justice interventions must be enhanced by collection of data on *how* different intervention designs enhance or undermine the social groups’ equality before and after access to justice. Further, moral arguments of justice interventions can be enhanced by better quantifying and comparing their economic and societal benefits, particularly where this can be done in such a way to demonstrate disproportionate impact for excluded social groups. Justice data collection and analysis, which employs broader societal data, as well as data generated from existing justice sector analytical methods, is a prerequisite for a “business case for justice”.^8^ An enhanced CBA APP, as described above, serves these objectives by improving the accuracy of justice sector resource allocation for maximum economic and societal outcomes while targeting society’s most vulnerable and excluded social groups.

Traditional CBAs commonly seek to weigh anticipated policy or intervention benefits against a policy reform or an intervention’s cost to determine overall societal benefit. CBAs demand a high volume of resources and time due to the many challenges of quantifying anticipated costs and benefits, idiosyncratic to the nature of policies and interventions in the diverse complexity of contexts in which they occur. Drawing on multiple sources of societal and justice process data in a systematic way, allows governments and stakeholders more exhaustive identification of interventions’ cost–benefits. It also enables systematic intervention comparison across a range of metrics beyond monetary benefits (such as crimes avoided and level of safety improvement) and to identify which interventions have greatest benefit for the most vulnerable social groups. Future utilisation of our enhanced CBA APP, which includes then tested heterogeneous impact component and future machine learning capacity, will enable significant contextual factors identified in previous CBAs to be tested in future intervention CBAs.

Our enhanced CBA APP confronts the human and material resource problem of repeated individual CBAs across different justice processes. Broader societal data (e.g., geographic, economic, demographic, climate, and security) as well as data specific to civil, criminal, and informal justice sector capacity can be retained and employed by the APP across (where appropriate) justice processes. Identified intervention costs are disaggregated by cost bearer (e.g., criminal justice system, local government, business, and society) and intervention outcomes (either positive or negative) may be qualified by moderators, including, via the use of governance and exclusion indicators, the social groups that benefit and the societal governance issues perceived as most demanding attention.

The enhanced CBA APP retention and future AI-driven vetting of data integrity component will enable re-vetting of data’s representativeness over time and alert researchers/users of the APP to potentially erroneous values (making suggestions about relevant contextual factors), and significant factors for data input narrowing. For development actors considering budget support or programmatic operations, enhanced capacity to determine the costs and benefits of policy and programming, particularly for the most vulnerable, enhances operational credibility, government process ownership, and development outcomes.

We know that since education and justice system investments constitute the most significant components of intangible capital, identifying optimal approaches to enhancing justice processes is critical for societal development. Our enhanced CBA APP takes the first important steps towards enabling government and stakeholder-led use of refined economic tools that incorporate artificial intelligence to advance our analytical capacity to drive these inclusive justice objectives. The APP we are continually developing is a direct response to the demand for economic evidence that respects the diversity in costs and benefits, and utilises developments in data science to improve the management and use of data. These steps in the development of the various modules defined above require testing to validate their implementation in the real world.

## Data availability statement

The simulated data supporting the conclusions of this article will be made available by the authors, without undue reservation.

## Ethics statement

The studies involving human participants were reviewed and approved by Human Research Ethics—Griffith University. Written informed consent to participate in this study was provided by the participants’ legal guardian/next of kin.

## Author contributions

MM and GW contributed to the conception and design of the study, developed the method outlined, developed the Smart CBT APP to analyse the data, and wrote major sections of the manuscript and its revisions. CM contributed to the conception of the study and the writing of major sections in the introduction and discussion. AV contributed to the development of the Smart CBT APP and coding. All authors contributed to the article and approved the submitted version.

## Conflict of interest

The authors declare that the research was conducted in the absence of any commercial or financial relationships that could be construed as a potential conflict of interest.

## Publisher’s note

All claims expressed in this article are solely those of the authors and do not necessarily represent those of their affiliated organizations, or those of the publisher, the editors and the reviewers. Any product that may be evaluated in this article, or claim that may be made by its manufacturer, is not guaranteed or endorsed by the publisher.

## Footnotes

^1^The three other ‘arenas’ of societal contestation associated with social group specific grievances surrounding exclusion include; access to political power, access to land and resources, and access to public services. See United Nations and World Bank (2018).

^2^See analytical approaches employed by the World Bank: https://www.worldbank.org/en/topic/governance/brief/justice-rights-and-public-safety.

^3^The current version of Smart CBA, with crime-related data as examples, can be found at: Manning et al. (2022). New examples are regulalry uploaded to demonstrate the capability of the tool to be adopted in different contexts. For access, please contact the lead author of this paper.

^4^Their consideration of CBA is specific to Legal Aid.

^5^See for example, Frölich and Melly (2010).

^6^The UN and World Bank cite a framework that breaks down the jurisdictional and functional elements of criminal justice processes that advance or undermine equality before the law.

^7^A measure of the relative harm from a crime or set of crimes that assist in the estimation of returns on investment from a given intervention. Refer to Curtis-Ham and Walton (2017) or an example of the application of a harm index.

^8^The ‘Business case for Justice’ was demanded by an agreed Joint Communique at the June 2019 Meeting of G7+ Justice Ministers in The Hague (Communique on file with authors who attended the conference).
