# Corrigendum: A method and app for measuring the heterogeneous costs and benefits of justice processes

**DOI:** 10.3389/fpsyg.2023.1281238

**Published:** 2023-10-30

**Authors:** Matthew Manning, Gabriel T. W. Wong, Christopher Mahony, Anushka Vidanage

**Affiliations:** ^1^City University of Hong Kong, Kowloon, Hong Kong SAR, China; ^2^Centre for Social Research and Methods, College of Arts and Social Sciences, Australian National University, Canberra, ACT, Australia; ^3^World Bank Group, Washington, DC, United States; ^4^Software Innovation Institute, College of Engineering and Computer Science, Australian National University, Acton, ACT, Australia

**Keywords:** justice reform, cost–benefit analysis, machine learning, data science, justice processes, heterogeneity

In the published article Manning et al. (2022) and Manning et al. (2018) were not cited because they were de-identified for review. A correction has now been made to Introduction, Paragraph 6. The corrected paragraph appears below:

“More recent developments have been undertaken by the authors of this paper (Manning et al., 2022), representing an extension of the above-mentioned MCBT, which begin to incorporate machine learning and artificial intelligence, including the development of an online CBA APP (Manning et al., 2022) as showcased in Manning et al. (2018). This APP takes important steps towards robust and time-sensitive analytical methods. The online CBA APP (currently in various stages of development), has been validated using a range of crime data,^3^ providing a framework with systematic data management capacity that enables user input support and EA. The online APP also includes a new heterogeneous component (which we describe and test here), that reveals and measures variations across social groups informing justice reform investment decisions that best manage and mitigate social group specific grievances while maximising economic consequence to society. We refer to this APP hereon as the “enhanced CBA APP”.

A correction has also been made to Method, Paragraph 2. The corrected paragraph appears below:

“**Figure 4** illustrates the enhanced CBA APP. In this study, we demonstrate three of the six interacting modules (Modules 1, 2, and 3). A full discussion of the six modules included in the enhanced CBA APP is provided by Manning et al. (2018).”

A correction has also been made to The benefits of the described CBA APP modules and next steps, Paragraph 1. The corrected paragraph appears below:

Presented above was a clear outline and test of *Modules* 1 to 3 of the enhanced CBA APP. The data driven capacity within the current version of the enhanced CBA APP can identify which justice processes and societal factors are most significant for the costs and benefits of processes specific to context. The current APP, therefore, is capable of accounting for macro variables like inflation, provision of best and worst-case scenarios, identification and accounting of data bias, proportion of costs borne per year, and effects on outcome, including outcomes specific to social groups, to context and to specific intervention elements. Manning et al. (2018) provide a detailed discussion on these elements. However, the current version of the enhanced CBA APP is capable of more than what we have presented here. Below we describe three additional modules that are currently in various stages of development, testing and implementation.

A correction has also been made to Footnote 3. The corrected footnote appears below:

“The current version of Smart CBA, with crime-related data as examples, can be found at: Manning et al. (2022). New examples are regularly uploaded to demonstrate the capability of the tool to be adopted in different contexts. For access, please contact the lead author of this paper.”

The authors apologize for these errors and state that this does not change the scientific conclusions of the article in any way. The original article has been updated.
